# A Systematic Review of the Use of T-Pattern and T-String Analysis (TPA) With Theme: An Analysis Using Mixed Methods and Data Mining Techniques

**DOI:** 10.3389/fpsyg.2022.943907

**Published:** 2022-07-22

**Authors:** María Consuelo Sáiz-Manzanares, Laura Alonso-Martínez, Raúl Marticorena-Sánchez

**Affiliations:** ^1^Area of Developmental and Educational Psychology, Department of Health Science, Facultad de Ciencias de la Salud, Universidad de Burgos, Burgos, Spain; ^2^Area of Didactics and School Organization, Department of Education Science, Facultad de Ciencias de la Educación, Universidad de Burgos, Burgos, Spain; ^3^Area of Computer Languages and Systems, Department of Computer Engineering, Escuela Politecnica Superior, Universidad de Burgos, Burgos, Spain

**Keywords:** behavioral structure, similarity, systematic review, THEME, T-Pattern, T-String, T-System

## Abstract

In recent years, research interest in human and non-human behavioral analysis has increased significantly. One key element in the resulting studies is the use of software that facilitates comparative analysis of behavioral patterns, such as using T-Pattern and T-String analysis -TPA- with THEME. Furthermore, all these studies use mixed methods research. Results from these studies have indicated a certain amount of similarity between the biological, temporal, and spatial patterns of human social interactions and the interactions between the contents of their constituent cells. TPA has become an important, widely-used technique in applied behavioral science research. The objectives of the current review were: (1) To identify the results of research over the last 4 years related to the concepts of T-Pattern, TPA, and THEME, since it is in this period in which more publications on these topics have been detected (2) To examine the key concepts and areas in the selected articles with respect to those concepts, applying data and text mining techniques. The results indicate that, over the last 4 years, 20% of the studies were laboratory focused with non-humans, 18% were in sports environments, 9% were in psychological therapy environments and 9% were in natural human contexts. There were also indications that TPA is beginning to be used in workplace environments, which is a very promising setting for future research in this area.

## Introduction

The T-System (Temporal System) is a method for analyzing patterns of events over time, often revealing patterns that may be hidden when using other methods of analysis. It refers to a set of temporal data (T-data), which is the accumulation of occurrences over a continuous period of observation (Magnusson, 2020a,b). According to Magnusson (2020a), the T-System indicates the similarity of measurement at a nanometer scale (measurements of physical and chemical properties of matter at scales of one millionth of a millimeter) and analysis of human and non-human behavioral patterns, analyzed in as microanalytical a manner as possible. T-data can be collected using multimedia encoders—which may be chips embedded in various tissues—that computerize the DNA or protein molecular data, transforming it into two-column text files that are processed via the THEME software (Magnusson, 2020a). The collected data are stored in separate files which in turn can be linked together and analyzed in combination as a multi-sample file within THEME (Magnusson, 2020c). THEME v. 6 Edu is free software that allows us to analyze the characteristics of the data and to represent the results as dendrograms and tree diagrams, among other functions (Arias-Pujol and Anguera, 2020). From this information analyzed in THEME, patterns are established based on a minimum number of occurrences found based on search parameters (Magnusson, 2020b). Patterns will repeat more frequently in a multi-sample file than in a single-sample file (Magnusson, 2020a). Data is collected using mixed methodologies that record qualitative data related to the event being studied and quantitative data linked to the frequency of occurrences of these events in real time (Magnusson, 2020c).

T-Patterns (temporal patterns) are segments of signals that are frequently repeated throughout the temporal signal sequence. For example, temporal signal sequences can be movements of the head, eyes, hand, etc. or of the body in general. T-Pattern detection was developed by Magnusson (1996, 2000, 2004, 2005, 2006, 2009, 2016, 2017, 2018, 2020a,b), who considered patterns to be a combination of events that invariantly follow each other in a given sequential and temporal order. They can also refer to patterns of behavior and habits in humans or non-humans. Detection of T-patterns in the T-System is relevant to understanding the relationship between human behavior and phenomena which occur at different levels of biological, temporal and spatial organization (Magnusson, 2020a). Physical strings containing spatial patterns are called T-Strings (Temporal strings, referring to strings in time) and are considered the latest addition to the T-system, for example, the analysis of patterns in the transformation and encoding of DNA into text (Magnusson, 2020b).

### Origin and Description of T-Pattern and T-String Analysis (TPA). Similarity Between Nano and Human Scales

The initial inspiration for the T-Pattern was based on classical theories such as Chomsky's (1957) homogeneity of the human genome for learning language, Skinner's (1969) operant conditioning behaviorism, and Tinbergen's (1963) ethological research relating the behavioral patterns of non-human species in their natural environments. These theories shared the need to understand how pre-established traits are established by the existence of behaviors that occur at the same time, follow each other, and appear with a certain frequency (Magnusson, 1981, 1983, 1989). The technological development of computer science, and especially artificial intelligence, has made it possible to draw lines between phenomena examined in psychological theories such as the computational theory of the mind, cellular-level processes and, in turn, computational algorithms (Magnusson, 2020a).

This mathematization of data facilitates the detection of formal and hidden patterns (referring to behavioral patterns—habits or behaviors—or motor behaviors—movements, stereotypies—that may be explicit or easily detectable, and others that require deeper analysis or different analysis in order to be revealed) and suggests artificial categories of certain temporal configurations (Magnusson, 2018). In particular, Heylighen (2016) it refers to Stigmergy as a universal coordination mechanism and its components and Collignon and Detrain (2020) analyze the relationship between self-organization and stigmergy. Specifically, the T-Pattern can be considered a hierarchical structure similar to a geometric object (akin to a fractal) which repeats itself at different scales in a single discrete dimension, is initially produced in real time, and exhibits similarities between its structures at spatial, temporal and biological levels (Magnusson, 2020b). These structures are characteristic of molecular organization such as genes and the corresponding 3D extension of their folded proteins (Magnusson, 2020a). In this way, the T-Pattern facilitates the detection of hidden structures that support human behaviors at the nanoscale level and allow them to be categorized, and is widely used in various scientific disciplines such as biology, computer engineering, psychology, pedagogy and mathematics (Casarrubea et al., 2018, 2020). Likewise, these findings contribute to the development of artificial intelligence when applied to the study of neuronal interactions (level 10^−6^) and are combined with applied statistics methods to promote the detection of effects produced by experimental variables (Nicol et al., 2015; Castañer et al., 2020).

The premise of detection in this interactive chain of behaviors is supported by the underlying stable T-Pattern structures (Arias-Pujol and Anguera, 2020; Cenni et al., 2020; Santoyo et al., 2020). T-Pattern analysis (TPA) is based on the use of algorithms that calculate temporal distances between behavioral codes and evaluate the critical interval that remains unchanged (Suárez et al., 2018; Santangelo et al., 2020). This pattern can also be used to decipher unfamiliar non-verbal interactions and to understand how they function and their meaning, as well as contributing to diagnosing individual and group behavioral traits with respect to the experimental conditions in which they occur (Magnusson, 2020b). Standardization of the analysis is serving as a methodological guide for an assessment of social interaction involving 32 European and US universities (Magnusson, 2018). Some of the limitations of TPA noted by Magnusson (2020b) are that the analysis is mainly performed on binary trees, which are inefficient for detecting higher-order structures; the significance is decided by the users controlling the programme and not by the algorithm; and each variant of the pattern can only be detected as a different pattern when it occurs a certain number of times (Portell et al., 2019; Gunst et al., 2020).

TPA allows for the analysis of similarity between the nanoscale and the human scale by encompassing spatio-temporal information from different levels of biological organization (Magnusson, 2018). Recently, advances in cellular and computational biology have allowed T-Strings (spatial T-Patterns in physical chains) to be analyzed using TPA, allowing analogies to be made between those T-String patterns and the information chains in cellular DNA that have remained stable for millions of years (Arias-Pujol and Anguera, 2020; Karcioglu and Bulut, 2021). These analyzes are helping us to understand the specific and joint tasks performed by organelles within each cell for cell survival, something which has been called “Cell City” (Magnusson, 2020a). The “Cell City” is a way of considering cellular organization made up of different organelles that perform various tasks to ensure the cell remains active. For instance, mitochondria can be thought of as similar to an energy company, the nucleus is the control center, similar to city hall, and the cell membrane resembles the boundaries of a city (Magnusson, 2020b). Ribosomes are made up of RNA and proteins and are the organelles responsible for protein synthesis by reading messenger RNA sequences and using the genetic code to translate those into amino acid sequences (Arias-Pujol and Anguera, 2020). The “Cell City” provides a way of thinking about the behavior of organisms or institutions (schools, factories, etc.) like proteins. Organizations instruct people how to process information, access it and encode it in their working memory so that they can perform whatever tasks are useful for the functioning of the city. The connections between the different institutions comprising different individuals is similar to the connections between neural networks and the different organs that condition behavior (Prat et al., 2019).

From this simile, an organelle can be represented as an entity or institution in the city, each with its own role, and organized interaction will ensure the functioning of the cellular metropolis (Magnusson, 2020b). It has been appreciated that connecting the processing chains between the organelles does not require great intellectual power other than defining the structures for systematic application of data operations that are similar and having a common simplified language to communicate effectively between the participating members of the “Cell City” (Magnusson, 2018). In contrast to the functioning of cellular organelles within the Cell City, the human brain has great intellectual power that could hinder synchronization and coordination between numerous individuals due to a lack of external chains controlling behavior through mass copying and distribution mechanisms (Casarrubea et al., 2019a; Casarrubea and Di Giovanni, 2020). This freedom of human decision-making in T-societies is changing and resembles the functioning of cellular organelles, as human behaviors are tending to become more homogenous globally as virtual connexions to the networks of mass societies are established. This situation promotes the development of increasingly similar social behaviors in what has been understood as globalization (Casarrubea et al., 2015).

Recent technological progress and advances in research support the similarities found between the behavioral and social structures that occur between proteins and human mass societies. Human organization tends to follow the patterns of cellular organization (Santoyo et al., 2020). In addition, there seems to be a similarity between the conversion of codes stored in RNA into DNA and the human capacity for information processing in problem solving or task solving in different situations (Magnusson, 2020b). Genetic structures are made up of purely informational strings and, in turn, the organization of human knowledge is largely underpinned by text processing (Portell et al., 2019; Prat et al., 2019; Camerino et al., 2020). These same analogies comparing the nanoscale functioning of the cell with the functioning of cities have previously been related to the gregarious behavior of insect groups (Merlet et al., 2005). The study of the underlying mathematical, physical and biological principles of T-societies applied to ethology showed that mass societies refer to structured animal groups of more than 10^4^ individuals working collectively to achieve a common goal, such as social insects and modern humans (Magnusson, 2020a).

Humans have advanced from nomadic lifestyles to missions of exploration on other planets, linked to eternal colonization and cellular expansion focused on the purpose of securing and enhancing the survival of the species (Casarrubea et al., 2018). It seems that human societies are fully connected and stem from nano-creatures (Magnusson, 2020a). Human specialization is considered unique among animal species, as text coding increasingly resembles the specialization of the various proteins involved in structural and organizational processes within an organism (Dejan et al., 2013). The specific tasks in each human cell are regulated by how they are encoded, and errors in transformation of information can lead to cellular malignancy that can be predicted, detected and prevented if we know the patterns of these behaviors (Nicol et al., 2015). Wars can be compared to cellular miscoding that causes cells to become malignant and invade other tissues, causing the destruction of the organism, while at the human scale, they can devastate the planet (Casarrubea et al., 2019b, 2021b). According to Magnusson (2020b), an analogy of the T-String with religions can also be seen by noting the importance of standardized texts, their distribution and copying to generate a pattern, which in this case, is the codification of the moral rules of conduct to be followed, and by noting that certain religious creeds become dominant over others by replacing existing codes with their own. All this makes us rethink human behavior by suggesting that war, overpopulation, global warming, diplomatic relations, and the control of resources, show that we tend to follow cellular patterns, as do the repetition of historical events that have occurred throughout evolution (Magnusson et al., 2016). As de La Mettrie (1748) explained in “The Machine Man,” this could lead us to ask ourselves whether we are just “robots” and that what we are doing is programming other structures similar to ours to repeat the patterns that have been standardized in our genetic code (Anguera et al., 2018).

Similarity from the nanoscale world to the human world started with linking from higher to lower levels in accordance with the discoveries that have been made (Magnusson, 2018). This premise does not indicate that a pattern corresponding to simpler data must always correspond to more complex data and vice versa, but rather that the discovery of similarity is considered to have come from the study of higher-level patterns because they were the data first accessed through natural observation (Santoyo et al., 2020). These similarities are related to the fact that the cell is part of the human body and is therefore one of the smallest structures that make up mass societies (Arias-Pujol and Anguera, 2020). Analogy is a valuable tool for knowledge and provides a new source of information on the current, rapid, dramatic changes in how humanity lives. Nevertheless, these studies also highlight that there is no simple evolutionary pathway linking the inner workings of cells and human behaviors (Casarrubea et al., 2019c).

### The Use of T-Pattern and T-String Analysis in Applied Research

After noting the similarity of human behavioral patterns analyzed in detail at the smallest possible scales of analysis similar to the structure of nanoscales in Temporal Partnership Analysis (TPA-T), the application of TPA in T-Society current research will now be explored. TPA-T is especially important in Behavioral Sciences because it facilitates the qualitative and quantitative informatisation of data by connecting information at the nanoscale with the human scale (Del Giacco et al., 2019). This extends the applicability of TPA to different disciplines ranging from genetics, proteomics, mass spectrometry, neuroscience, psychology, ethology, biology and mathematics to religious science (Magnusson, 2018).

The systematic review by Casarrubea et al. (2015) summarized application of the T-Pattern in non-human and human behavior. At the no-human level, numerous studies have used these processes to compare and evaluate stereotypical behaviors in captive and wild animals. For example, starlings living in captivity showed more inactivity, greater route-finding and escape behaviors, and less impulsivity in their decisions than those not living in captivity (Feenders and Bateson, 2012, 2013). Using TPA, differences were also found in feeding behaviors of birds and parasitic animals in relation to available resources and nutrient foraging, with patterns indicating increased stress in the absence of these resources (Merlet et al., 2005; Hemerik et al., 2006). TPA was also used to find how Embioptera (an insect) marked its territory and defended it from potential invaders (Dejan et al., 2013). In addition, experimental animal studies have been performed in order to understand the origin of human behaviors. In studies with mice it was discovered that there were prior genetic factors related to Obsessive Compulsive Disorder, as the mice responded differently even though they had all been pharmacologically stimulated (de Haas et al., 2012). In rats, the influence of prior learning was studied, showing patterns of improvement in task performance (Casarrubea et al., 2015, 2021c) and by investigating the structural functional patterns of olfactory bulb cells in these animals, it was discovered that the physiological properties of the neural system form part of the basis for encoding sensory information (Nicol et al., 2015). In addition, the T-Pattern has allowed the study of patterns associated with vomiting in shrews (Horn et al., 2013), the swimming patterns of cod (Jonsson et al., 2010) and the underlying nature of group actions in wolves (Yachmennikova and Poyarkov, 2011). Another study by Casarrubea et al. (2021a) analyzed the effect of smoking on anxiety-like behavior in rats. They used a quantitative and multivariate T-pattern analysis. The latter revealed that rats chronically treated with nicotine continued to show anxiety-like behavior after nicotine challenge.

TPA has also been applied to humans in numerous research studies, starting with examining patterns of various mental illnesses and disorders. The study by Warreyn et al. (2007) analyzed the behaviors of children with autism spectrum disorder, showing that the onset of declarative behavior was different and characterized by intermittent attention performance between the object and the person; for example, when pointing, children with autism looked at the pointing finger instead of the object being pointed at. These children exhibited less demand skills and slower task tracking performance. In the study by Masunami et al. (2009), children with Attention Deficit Hyperactivity Disorder (ADHD) exhibited differences in decision-making compared to children without ADHD. Children with ADHD exhibited lower T-Patterns in response to punishments, indicating that they paid less attention to punishments than children without ADHD. Increased detection of different T-Pattern responses has been found in people with schizophrenia taking clozapine (Lyon and Kemp, 2004). Furthermore, this increase in T-Patterns was shown to be exacerbated in people who engaged in self-harm (Sandman et al., 2012). In the field of professional sports, Pic and Jonsson (2021) found higher percentages of T-Pattern recording in boxers who won compared to boxers who lost. From these patterns it was found that winners exhibited greater complexity in decision-making and tended to fight in the area closer to the center of the ring. Along similar lines, Muñoz-Arroyave et al. (2021) took a multidimensional perspective (decisional, relational and energetic) to study the interpersonal relationships established by boys and girls in the traditional game of Elbow Tag. The authors applied classification trees and found that the T-pattern analyzes between girls and boys in a mixed group were unequal. This difference was mainly due to decision making (subrole variable), which had much greater predictive power than the energetic variables. Martín-Martínez et al. (2021) analyzed players' decision making in Marro (a traditional game) using a multimodal approach with Crosstabs, adjusted residuals (AR), classification trees (Chaid model) and T-pattern analysis (TPA). The results indicated that the use of different methodologies in the analysis provided data for an individualized study of player behavior. Similarly, Pic et al. (2018) studied the triadic relationship in the practice of motor games. They used the detection of behavioral patterns not visible to the naked eye, which were analyzed with Theme software to detect temporal regularities in the order of occurrence of events. They found that the temporal location of the motor responses in the triad games was not random.

Other influential studies have focused on analyzing the relationships of T-Patterns with different mental conditions and intra-individual and intergroup processing. These studies have focused on various topics, such as social interaction in infants (Magnusson, 2020a), pervasive growth disorders (Willemsen-Swinkels et al., 2000), behavioral symptoms of dementia (Woods and Yefimova, 2014), self-directed speech and non-verbal behavior (Kuvalja et al., 2014), language and behavior patterns (Blanchet et al., 2005), stressors and routine tasks (Su et al., 2013), effectiveness and interactions in teams (Zijlstra et al., 2012), human-cat interactions (Wedl et al., 2011), and human-robot interactions (Jonsson and Thorisson, 2010).

### The Use of T-Patterning and T-String Analysis With Theme Software in Mixed-Methods Research

The use of mixed methods in psychotherapeutic research, including TPA with THEME, has increased in recent years, based on the need to adapt to and capture changes within the continuum of therapies (Voutilainen et al., 2018). These tools allow behavioral data to be analyzed, collected and recorded from the beginning to the end of the session, combining qualitative and quantitative data together (Bartholomew and Lockard, 2018). This means that data may have been obtained using a variety of techniques such as observation, narrative, interviews, surveys, physiological testing and a range of other data taken as the therapeutic intervention is being conducted (Voutilainen et al., 2018). Linking these data to patterns allows numerous behavioral coding matrices to be created that facilitate methodological confrontation by promoting the use of mixed methodologies (Roberts and Allen, 2019). In the study by Arias-Pujol and Anguera (2020), these methodologies were considered in analyzing the communication strategies used by a psychotherapist with adolescents before, during and after therapy. Through TPA, patterns were isolated that promoted the good functioning of the therapy; these were related to the mental processes that occurred during the sessions, with the most successful therapies being those in which the therapist used interrogative phrases and paraphrases at the beginning and questioning and mentalization techniques at the end. The results from TPA were the same as those found using two other techniques (delayed sequence and polar coordinates) in the same study.

TPA has also been used in education, for instance in a study by Suárez et al. (2018) with six primary school teachers, it was used to demonstrate that the teachers' skills related to alphabetic knowledge and phonological awareness were used less frequently in the classroom than expected, with fewer than 50% of the teachers following the recommendations of the National Reading Panel. The study by Prat et al. (2019) used pattern analysis to evaluate educational interventions based on a pedagogical model of personal and social responsibility. This study made it possible to evaluate the emergent behaviors of each educator, comparing the initial, and final self-assessment of a teacher using traditional methodology with another using innovative methodology. TPA showed that the pedagogical performance of the teachers and the self-evaluations of their students were more positive if the educator used the innovative methodology. This innovative methodology focused on awareness, responsibility for task performance, group meetings and self-assessment. Moreover, analyzing the stability and change of T-Patterns, Santoyo et al. (2020) linked task persistence in preschool children with social interaction between them and their teachers. The study found that teachers attempted to redirect behavior and follow alternative loops to engage children's on-task attention and avoid distracting behaviors, those who did so most effectively consistently responded to children's social signals. Pic et al. (2021) analyzed the play of 23 children aged 12–13 through mixed approaches using TPA. They showed that girls had greater role variability in triadic motor play than boys. Gender roles were also found to influence and bias decision-making, with girls more frequently resorting to peer-release behaviors during play than boys.

Applied research studying the use of human behavioral analysis scales in small measurements or micro-measurements, similar to nanoscales, will foreseeably help in the understanding of behavioral patterns. Likewise, the use of predictive algorithms will facilitate detection of possible patterns of human behavior in different situations. Content classification is important for the creation of models to assess and analyze repeated sequences of behaviors. However, in recent years, the use of these analytical techniques have spread to contexts other than the original field of genetics. This systematic review therefore addresses the challenge of understanding the application of the multivariate approach based on the use of TPA through THEME in different contexts and groups. Mixed research methods were used for the analysis, applying data mining and text mining techniques. This systematic review methodology allows for an analysis which can adapt to different focuses of research interest (Sáiz-Manzanares et al., 2020).

The objectives of this study were:

(1) To identify the results of research in the last four years related to the concepts of THEME, T-Patterns, and T-Strings, applied to human and non-human behavioral analysis.
(2) To apply data mining techniques to analyze the results from studies related to the concepts of THEME, T-Patterns, and T-Strings, applied to human and non-human behavioral analysis.
(3) To apply text mining techniques to analyze the results from studies related to the concepts of THEME, T-Patterns, and T-Strings, applied to human and non-human behavioral analysis.

## Materials and Methods

### Design

A mixed research methodology was used. Firstly, a descriptive design was applied in which the articles were analyzed quantitatively, using data mining and text mining techniques. Secondly, an explanatory design was applied in which the articles were analyzed qualitatively using network analysis techniques with the qualitative analysis programme Atlas.ti v. 9.

### Sample

**Figure 1:**
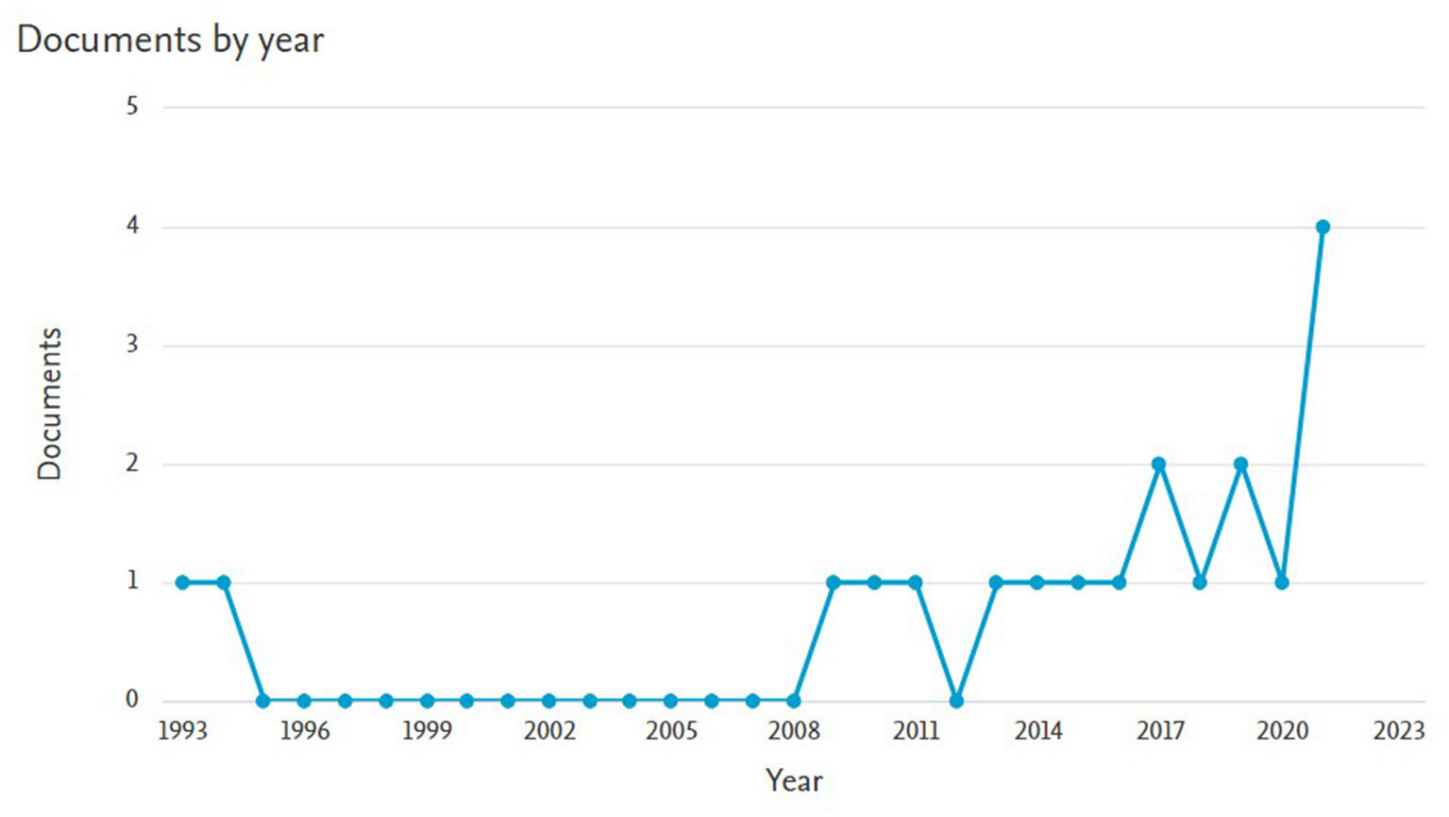
Records found in Scopus for the keyword “THEME TPA.”

**Figure 2:**
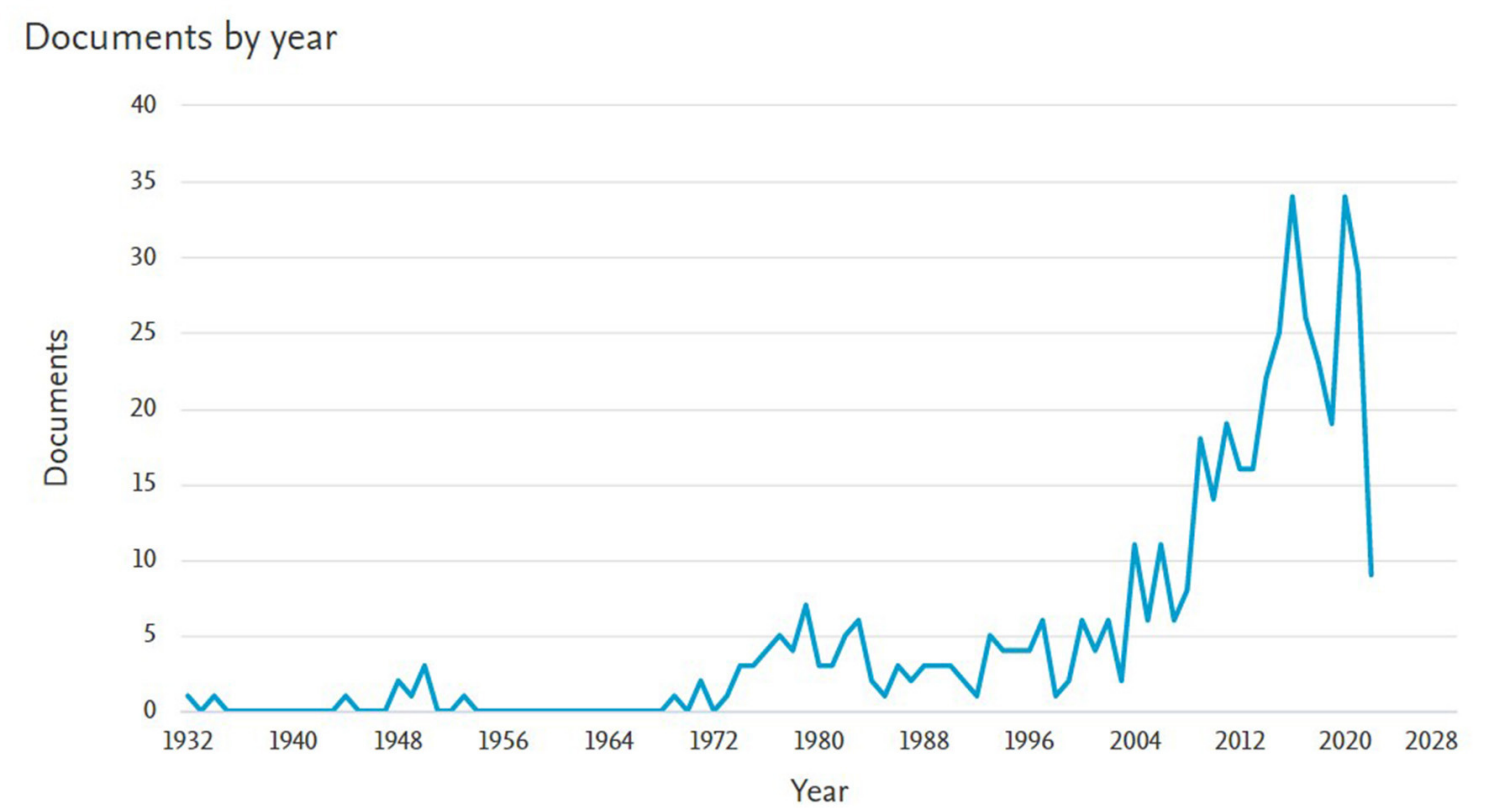
Records found in Scopus for the keyword “T-Pattern.”

**Figure 3:**
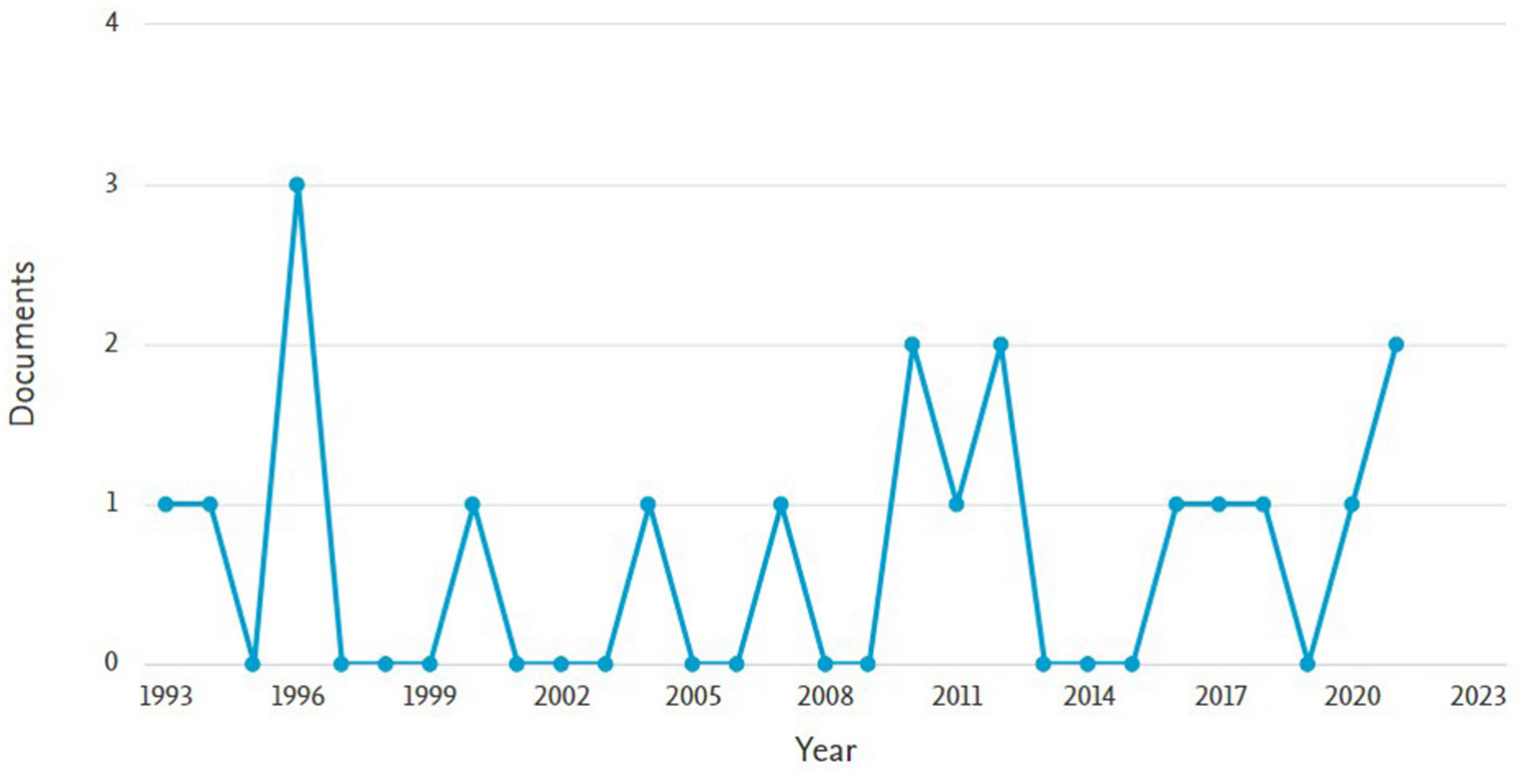
Records found in Scopus for the keyword “T-String.”

A literature review was performed using the keywords “THEME TPA,” “T-Pattern,” and “T-String.” Joint analysis of the three keywords did not yield any results, hence an individual search was performed for each word individually. “THEME TPA” produced 19 documents from 1993 to 2021. Searching for “T-Pattern” produced 467 documents from 1932 to 2022. For “T-String” 19 records were found from 1993 to 2020. The searches were performed using Scopus and Web of Science databases. Figure 1 shows the search results for the keyword “THEME TPA,” Figure 2 shows the search results for the keyword “T-Pattern” and Figure 3 shows the search results for the keyword “T-String.”

The knowledge areas for the documents were also examined for each keyword (see Table 1).

**Table 1:** Percentages of document type by keyword.

**Type of document**	**Keyword**		
	**THEME TPA**	**T-Pattern**	**T-String**
Neuroscience	8.1%	8.9%	3.4%
Multidisciplinary	–	–	3.4%
Biochemistry	8.1%	7.3%	3.4%
Agriculture	–	5.0%	3.4%
Social Sciences	13.5%	3.7%	6.9%
Physics and astronomy	–	–	6.9%
Psychology	10.8%	9.7%	6.9%
Medicine	24.3%	24.4%	10.3%
Mathematics	–	–	27.6%
Computer Science	2.7%	7.5%	27.6%
Engineering	5.4%	5.8%	–
Pharmacology	5.4%	3.8%	–
Other	10.8%	19.0%	–
Health Professions	–	4.9%	–
Nursing	8.1%	–	–
Marketing	2.7%	–	–

### Instruments

Scopus and Web of Science databases were used to perform the searches.

### Procedure

Firstly, a literature search was performed in the aforementioned scientific databases. The search criteria used were “THEME TPA,” “T-Pattern,” and “T-String.” The inclusion criteria were keywords in the period from 2018 to 2021, in the disciplines of psychology, medicine, neuroscience, social sciences and computer Science, other articles were excluded. The reason for this was that the highest proportion of publications including the keywords were published during this time and in these disciplines. Based on these criteria, 30 documents were found, 7 for “THEME TPA,” 21 for “T-Pattern,” and 2 for “T-String.”

**Figure 4:**
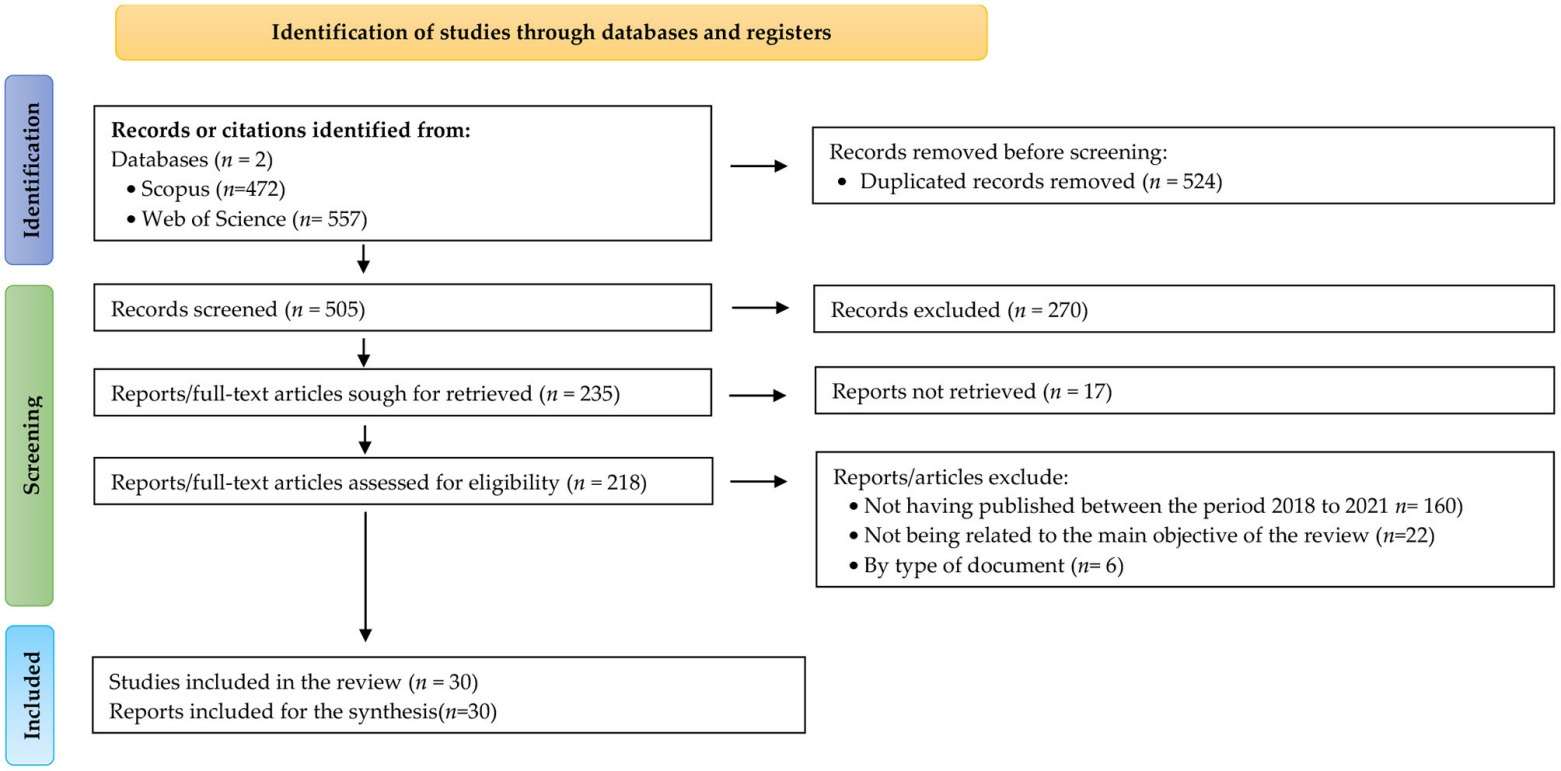
PRISMA flow chart of the selection processes for the articles included in the study following the PRISMA 2020 design from Page et al. (2021).

Two types of study were then performed. Firstly, a quantitative analysis was conducted by classifying the articles according to the following criteria: keyword, year of publication and study objective. Then data mining and text mining techniques were applied to analyze the results of that classification. Secondly, a qualitative study of the articles was performed to identify the concepts used in the 30 articles, classifying them by category and applying network analysis (see Figure 4).

### Data Analysis

The following techniques were used for the quantitative study in pursuit of the objectives: descriptive statistics techniques (frequency and percentage analysis) and unsupervised learning data mining techniques, specifically clustering using the k-means algorithm. The statistical package SPSS v.28 (IBM, 2022) was used for this purpose. In addition, Orange v.3.30.2 (Orange, 2022) was used to visualize the results, and Atlas.ti. (2020) was used to perform the text mining analysis. For the second, qualitative study, frequency recording, co-occurrence analysis and network analysis were applied, using the data management, processing and visualization software Atlas.ti. v9 (Atlas.ti., 2020).

## Results

### Quantitative Analysis of Articles

#### Classification of Items

The first objective concerned the analysis of research on the concepts of THEME TPA, T-Pattern, T-String with respect to behavioral, human health and social analysis. Studies including these three traceability references during the time interval from 2018 to 2020 were checked for 30 papers. Subsequently, these studies were sorted by year of publication and categorized according to the group they referred to (1 = THEME TPA, 2 = T-Pattern, 3 = T-String). The articles were then classified according to knowledge area, with ten reference criteria for classifying the research field (C1 = Gamification research; C2 = Sports research; C3 = Genetics research; C4 = Research in psychological therapies; C5 = Research in human behavioral analysis in natural contexts; C6 = Research in non-human behavior analysis in the laboratory; C7 = Research in human behavioral analysis in educational contexts; C8 = Research in human behavioral analysis with pharmacological consequences; C9 = Research on human behavioral analysis in laboratory settings; and C10 = Research on human behavioral analysis in occupational settings). The categorization of the individual studies and the frequencies are given in Table S1 (Supplemental Material). The percentage per category is shown in Figure 4. Furthermore, Table 2 shows the relationship of the selected articles with respect to the category of type of research and the intervention group (1 = Secondary school students; 2 = Athletes; 3 = Human genetic material; 4 = Rats; 5 = Humans; 6 = University students; 7 = Non-human primates; 8 = Preschool students).

**Table 2:** Classification of articles.

**Article**	**Intervention group**	**Year**	**Cluster**	**C1**	**C2**	**C3**	**C4**	**C5**	**C6**	**C7**	**C8**	**C9**	**C10**
A_1	1	2021	1	1				1					
A_2	2	2021	1		1			1					
A_3	3	2021	1			1							
A_4	4	2021	1						1				
A_5	4	2021	1				1						
A_6	5	2020	1							1			
A_7	5	2020	1							1			
A_8	6	2020	1							1			
A_9	2	2020	1								1		
A_10	5	2020	1		1								
A_10	7	2020	1						1				
A_11	5	2020	1		1								
A_11	7	2020	1						1				
A_12	8	2020	1							1			
A_13	6	2020	1							1			
A_14	2	2020	1		1								
A_15	4	2020	1						1				
A_16	2	2020	1		1								
A_17	2	2020	1		1								
A_18	2	2020	1		1								
A_19	4	2019	2						1				
A_20	4	2019	2						1				
A_21	5	2021	1						1				
A_21	7	2021	1								1		
A_22	3	2021	1			1							
A_23	5	2018	2					1					
A_24	5	2020	1				1						
A_25	5	2018	2									1	
A_26	5	2018	3	1	1	1	1	1	1	1	1	1	
A_27	5	2019	2										1
A_28	8	2018	2							1			
A_29	4	2019	2						1				
A_30	5	2019	2				1					1	

*C1, Research in gamification; C2, Research in sport; C3, Research in genetics; C4, Research in psychological therapies; C5, Research in human behavioral analysis in natural contexts; C6, Research in non-human behavioral analysis in laboratories; C7, Human behavioral analysis in educational contexts; C8, Human behavioral analysis with pharmacological consequences; C9, Research in human behavioral analysis in laboratories; C10, Research in human behavioral analysis in work contexts*.

**Figure 5:**
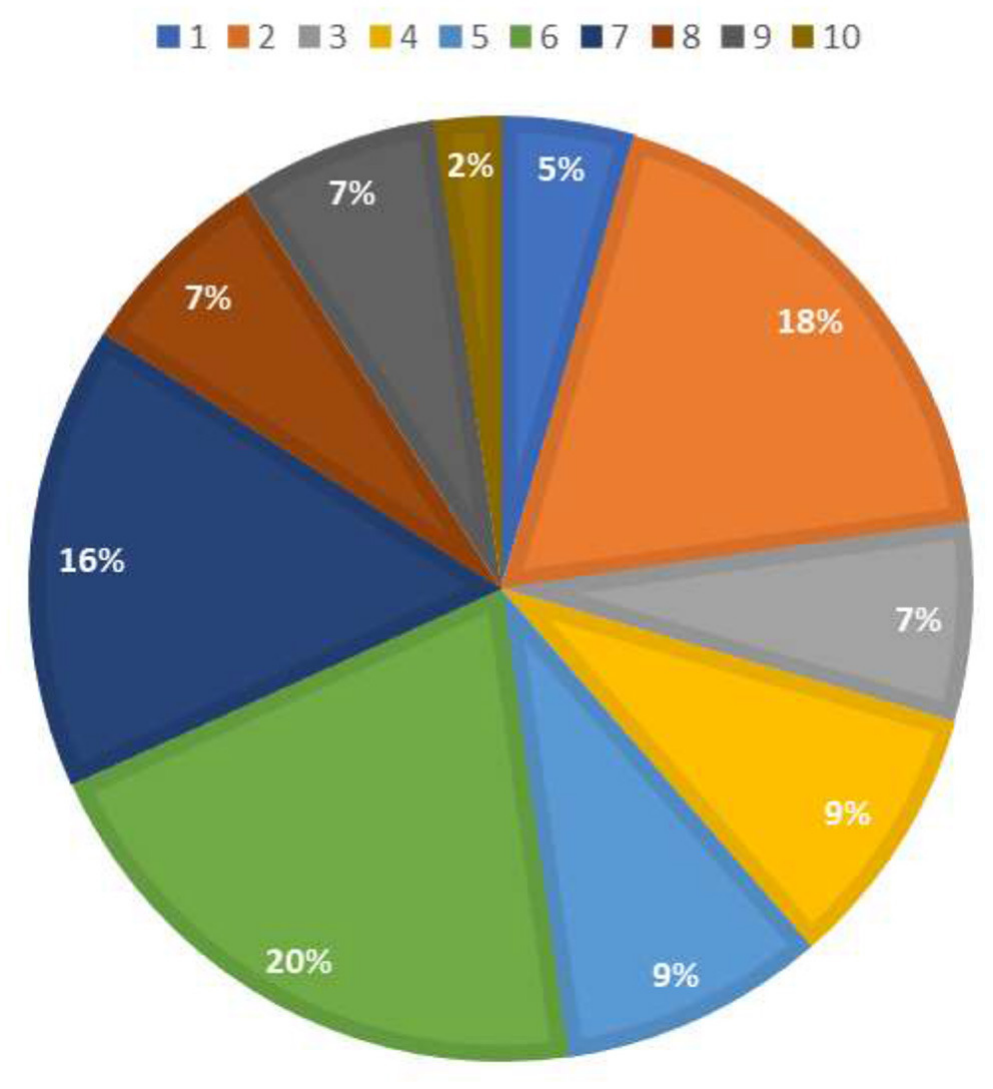
Percentage of studies in the period 2018–2021 by classification category. Note: 1 = C1. Research in gamification; 2 = C2. Research in sport; 3 = C3. Research in genetics; 4 = C4. Research in psychological therapies; 5 = C5. Research in human behavioral analysis in natural contexts; 6 = C6. Research in non-human behavioral analysis in laboratories; 7 = C7. Research in human behavioral analysis in educational contexts; 8 = C8. Research in human behavioral analysis with pharmacological consequences; 9 = C9. Research in human behavioral analysis in laboratories; 10 = C.10. Research in human behavioral analysis in work contexts.

In summary, the types of research with the greatest representation in pattern analysis with THEME software since 2018 were: 20% in non-human research in laboratory contexts, 18% in research in sports contexts, 16% in human behavioral analysis research in educational contexts, and 9% in psychological therapy research and human behavioral analysis research in natural contexts (see Figure 5).

#### Application of Data Mining Techniques

In pursuit of the second study objective, the *k-means* clustering technique was first applied using SPSS v.28 (IBM, 2022) to the frequency data matrix (Table 2). Three clusters were found, the distribution of which with respect to the categorization criteria of the articles is shown in Table 3. The number of the cluster found for each article is included in Table 2 in order to more clearly show the assignment relationship.

**Table 3:** Initial and final cluster centres.

**Classification criteria**	**Initial clusters**			**Final clusters**		
	**1**	**2**	**3**	**1**	**2**	**3**
	***n* = 28**	***n* = 1**	***n* = 4**	***n* = 28**	***n* = 1**	***n* = 4**
C1	1	1	0	0	1	0
C2	0	1	0	0	1	0
C3	0	1	0	0	1	0
C4	0	1	1	0	1	1
C5	1	1	0	0	1	0
C6	0	1	0	0	1	0
C7	0	1	0	0	1	0
C8	0	1	0	0	1	0
C9	0	1	1	0	1	1
C10	0	0	0	0	0	0

*C1, Research in gamification; C2, Research in sport; C3, Research in genetics; C4, Research in psychological therapies; C5, Research in human behavioral analysis in natural contexts; C6, Research in non-human behavioral analysis in laboratories; C7, Human behavioral analysis in educational contexts; C8, Human behavioral analysis of pharmacological consequence; C9, Research in human behavioral analysis in laboratories; C10, Research in human behavioral analysis in work contexts*.

The classification criteria C4 (Research in psychological therapies) and C9 (Research in human behavioral analysis in the laboratory) exhibited atypical values of belonging to two clusters. This can be explained by the simultaneous categorization of the articles into several categories, specifically article 26. Therefore, it could be reduced to a single category, which would be “Research in psychological therapies.”

**Figure 6:**
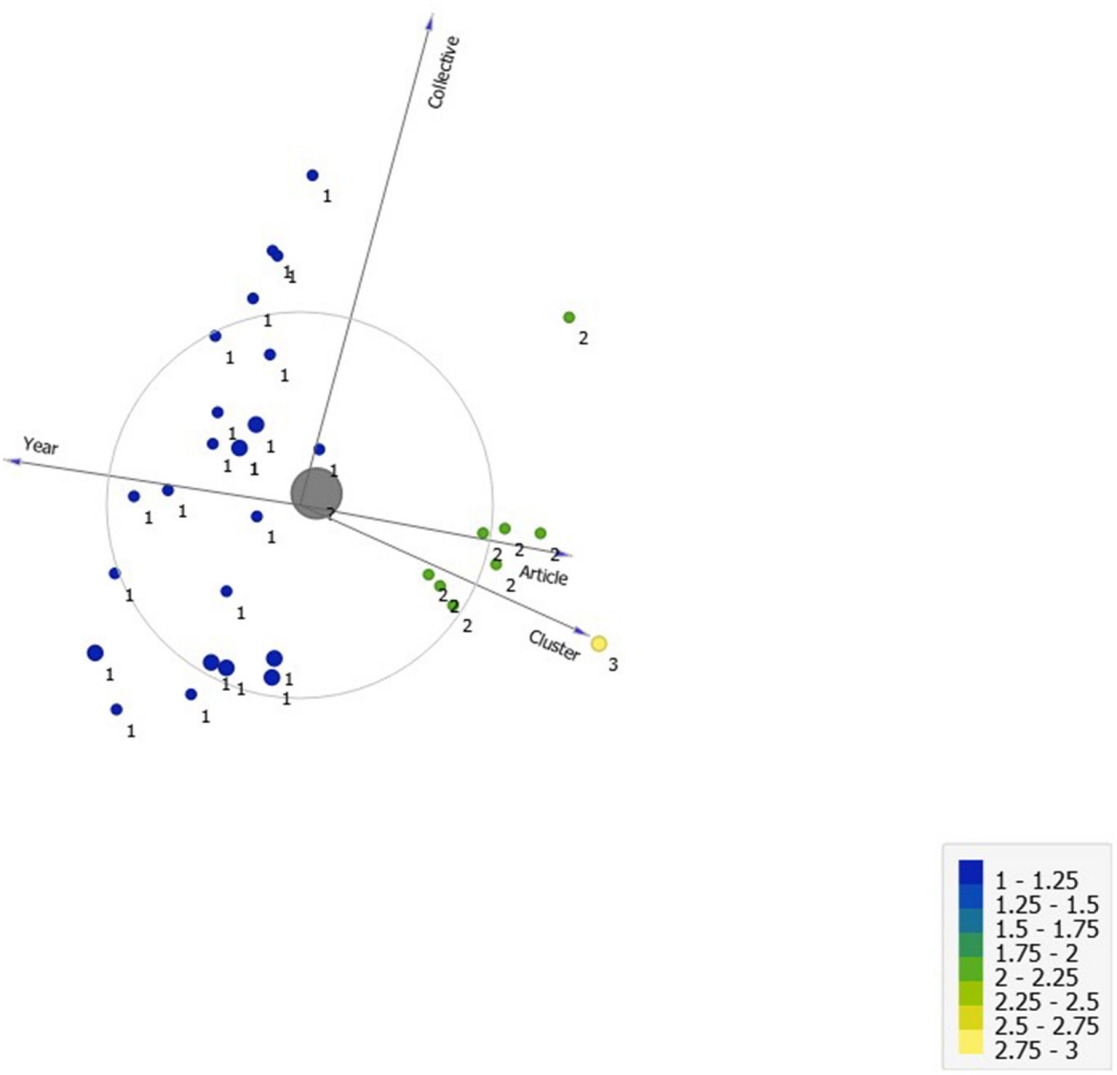
Distribution of the groupings in the categories established in the articles in a principal components analysis.

Subsequently, the results of the application of the *k-means* clustering technique were visualized with Orange Software v.3.30 (Orange, 2022). Figure 6 shows the distribution of the clusters with respect to the categorization. As the final cluster centers show, Cluster 2 includes all categories except C10 (Research in human behavioral analysis in work contexts). Likewise, Cluster 3 specifically includes C4 (Research in psychological therapies) and C9 (Research in human behavioral analysis in laboratory settings). Those criteria could be reduced to a single category, “Research in psychological therapies.”

#### Application of Text Mining Techniques

**Figure 7:**
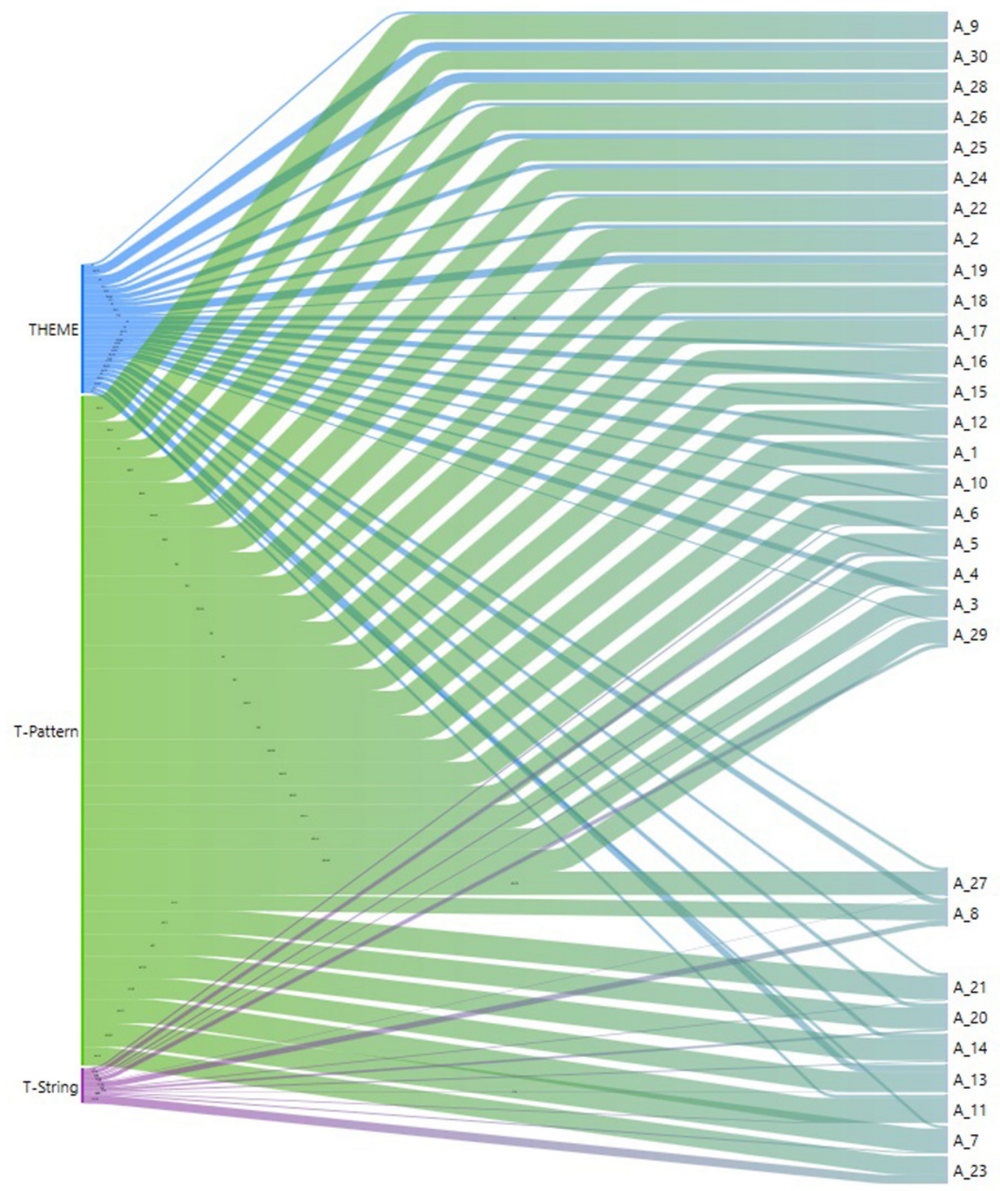
Sankey diagram for keywords per document.

In consideration of the second study objective, the information in the articles was analyzed by applying text mining using Atlas.ti v. 9 (Atlas.ti., 2020). A total of 3,990 keywords were recorded; THEME TPA *n* = 614, T-Pattern *n* = 3,209, T-String *n* = 167. A Sankey diagram of the binarized data with respect to the analysis of keywords per document is shown in Figure 7. This information may be very useful as it directly relates the document to the keyword.

**Figure 8:**
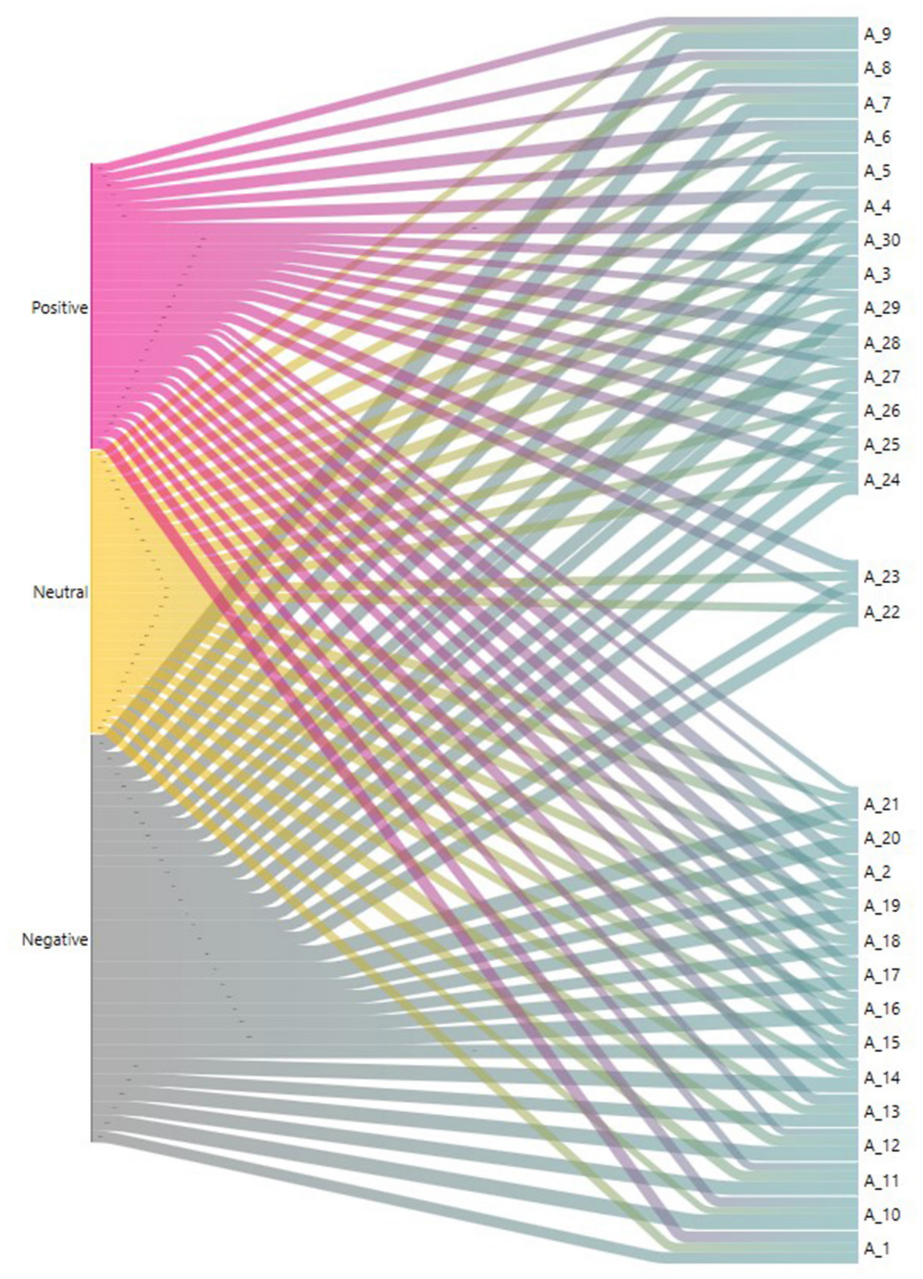
Sankey plot of the categorization of key (positive-negative-neutral) sentences per document.

In addition, sentiment analysis categorization was applied and a total of 16,590 categorizations were made, of which *n* = 4,856 were positive sentiments (sentences without negative particles), *n* = 6,942 were negative (sentences that include some negative particles), and *n* = 4,792 were neutral (sentences that include neither positive nor negative particles). Figure 8 shows a Sankey plot of the binarized data with the analysis of the sentiment categories per document. This information may also be useful as it provides information on possible study conclusions.

### Application of Qualitative Analysis

In pursuit of the third objective, co-occurrences between the selected documents and the applied categorization codes were analyzed. This analysis may be of great use to the researcher in terms of selecting the documents with respect to the keywords (THEME TPA, T-Pattern, T-String), the selected grouping criteria (C1, C2, C4, C5, C6, C7, C10)—although criteria C2, C8, and C9 were eliminated as they were at very low frequencies—and the type of sentences (positive, neutral and negative), see Table 4. Researchers may also find it useful as it provides information on the type of research with THEME and TPA in different fields, the frequencies of the keywords, and the type of sentences for each of the articles analyzed. For example, article 3 (A_3) refers to research in the field of human behavioral analysis in natural contexts, it has a frequency of 48 positive sentences, 61 negative sentences, and 53 neutral sentences. It also includes the keywords THEME TPA, T-Pattern and T-String with a frequency of 37,101 and 3, respectively.

**Table 4:** Analysis of document-coding criteria co-occurrence.

**Articles**	**Categorization criteria**												
	**C1**	**C2**	**C4**	**C5**	**C6**	**C7**	**C10**	**N**	**Neu**	**P**	**THEME**	**T-Pattern**	**T-String**
A_1	34	0	0	10	4	2	0	72	58	72	4	19	0
A_2	34	0	0	10	4	2	0	72	58	72	4	19	0
A_3	0	0	0	31	3	0	0	61	53	48	37	101	3
A_4	0	1	14	8	17	0	0	51	38	52	4	46	2
A_5	0	2	1	13	15	0	1	66	48	43	11	44	6
A_6	0	1	14	8	17	0	0	51	38	52	4	46	2
A_7	0	0	0	16	3	0	1	28	20	16	4	26	1
A_8	0	0	0	104	11	0	0	119	70	73	38	86	23
A_9	1	0	0	8	4	0	0	103	40	52	4	41	0
A_10	9	0	0	6	2	0	0	139	63	92	6	17	0
A_11	0	0	0	10	4	0	1	71	53	40	6	38	1
A_12	0	0	0	16	9	0	0	85	60	39	6	39	0
A_13	2	1	0	8	0	8	0	99	72	83	23	52	1
A_14	0	2	0	23	3	0	2	121	63	70	9	59	2
A_15	0	7	0	10	1	2	0	100	69	69	7	27	0
A_16	0	1	0	11	8	0	0	63	39	28	5	27	0
A_17	1	1	0	18	1	1	0	108	69	74	5	27	0
A_18	0	1	0	4	0	0	0	72	58	72	2	20	0
A_19	0	0	4	2	1	0	0	111	81	77	6	11	0
A_20	0	0	0	12	21	0	0	64	41	20	24	135	11
A_21	0	2	0	9	20	0	0	82	47	34	5	37	1
A_22	1	1	0	45	18	1	1	85	53	60	9	64	0
A_23	0	0	0	37	1	0	0	74	57	73	0	70	33
A_24	3	0	0	20	0	0	0	213	154	186	11	37	0
A_25	0	0	0	4	0	1	0	167	104	118	18	58	0
A_26	1	1	0	45	18	1	1	85	53	60	9	64	0
A_27	0	7	0	6	1	0	0	117	112	72	11	52	1
A_28	0	0	0	7	0	8	0	103	85	112	14	19	0
A_29	0	3	0	10	21	0	0	48	39	24	4	41	5
A_30	0	0	0	18	0	0	0	129	112	130	2	3	0

*C1, Research in gamification; C2, Research in sport; C4, Research in psychological therapies; C5, Research in human behavioral analysis in natural contexts; C6, Research in laboratory non-human behavioral analysis; C7, Human behavioral analysis in educational contexts; C10, Research in human behavioral analysis in work contexts. Criteria C2, C8, and C9 were removed as they were at very low frequencies. N, Negative; Neu, Neutral; P, Positive*.

**Figure 9:**
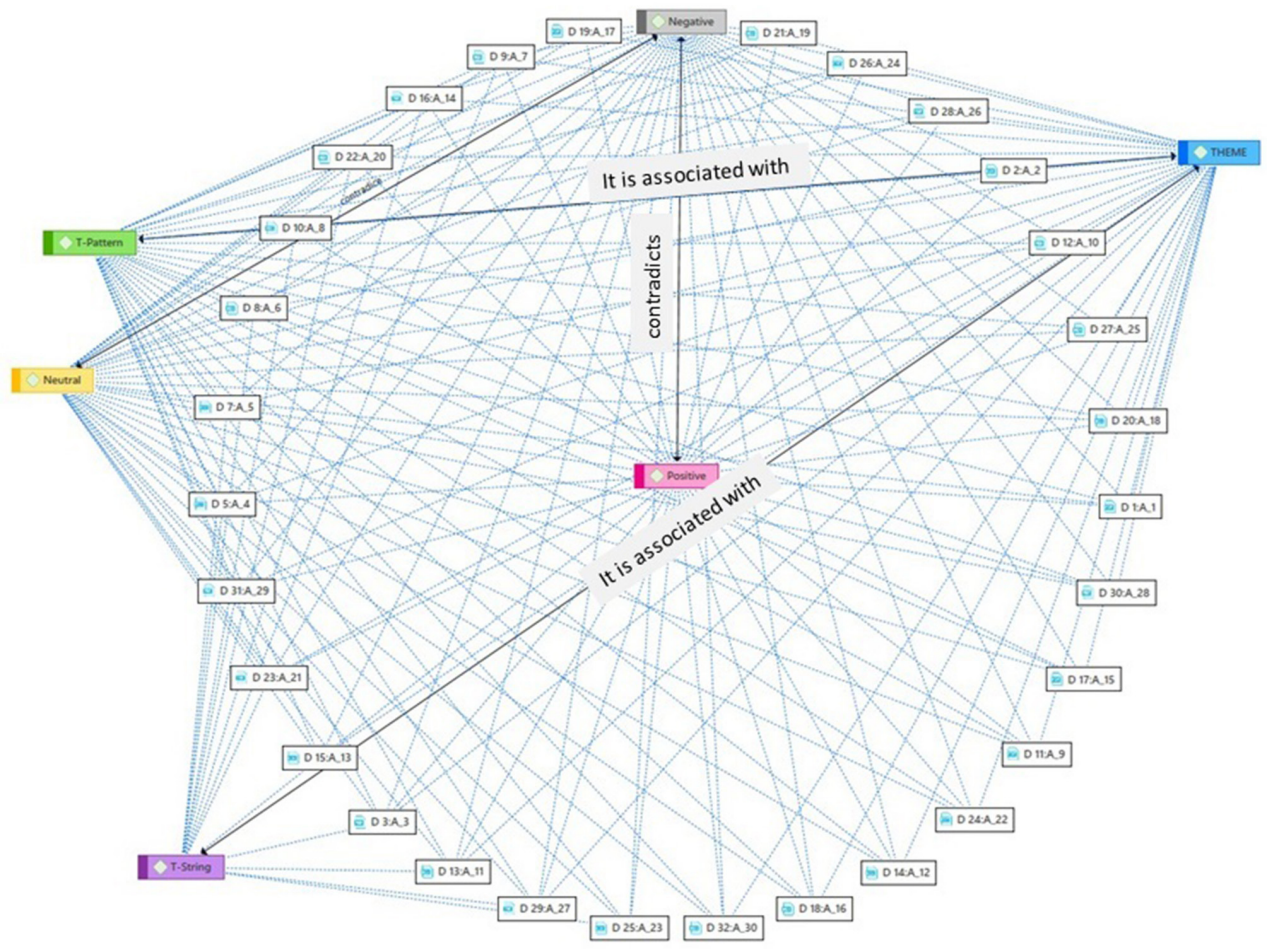
Network analysis of documents and categorization criteria.

Network analysis was then applied to the selected documents and to the categorization of the documents by type of research and categorization of sentences (positive-negative-neutral). To that end, an orthogonal-radial analysis was performed in which the relationships of each document with the different codes applied can be checked (see Figure 9). This visualization may be of great use to the researcher as it makes it easy to choose information according to the object of study graphically and interactively.

**Figure 10:**
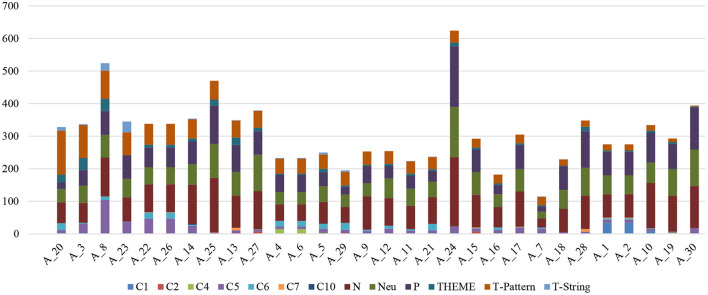
Ranking of the selected articles with respect to the categorization criteria from the T-Pattern reference.

Finally, Figure 10 presents the categorized relationship of the selected articles taking as a reference the frequency of use of T-Pattern with respect to the categorization criteria and the other keywords (THEME and T-String). This relationship allows the researcher to select the article or articles that best fit the object of research related to the use of THEME, T-Pattern and T-String.

## Conclusions

Using THEME to find behavioral patterns in humans and non-humans is an important asset in the understanding of behavior (Magnusson, 2020b). Moreover, incorporating artificial intelligence techniques facilitates relational analysis between human-scale behavior and cellular organization and functions (Anguera et al., 2018; Casarrubea et al., 2018; Arias-Pujol and Anguera, 2020; Magnusson, 2020a,b). Likewise, the use of mixed research methodology applied to TPA allows the analysis of human behavioral traits that are supported by the detected patterns, among other things (Bartholomew and Lockard, 2018; Roberts and Allen, 2019). This makes it easier to study large amounts of behavioral information, and more significantly, to draw conclusions based on algorithmic measurement. Similarly, approaches that have traditionally been applied in the context of genetic research are being generalized to research in psychological therapy (Bartholomew and Lockard, 2018; Voutilainen et al., 2018; Arias-Pujol and Anguera, 2020; Brill and Schwab, 2020; Gutiérrez-Santiago et al., 2020; Prieto-Lage et al., 2020a,b; Santoyo et al., 2020) and in education (Suárez et al., 2018; Prat et al., 2019).

The specific results from the present study show that, in the last 4 years, the studies that used THEME software to perform TPA were distributed as follows: 20% in laboratory research contexts with non-humans, 18% in behavioral analysis settings in the field of sports, 16% in educational contexts, 9% in therapeutic intervention contexts in psychology, and 9% in natural human contexts. This analysis methodology is also beginning to be used in work environments, although so far in only 2% of the studies. Work is a promising area for behavioral analysis, as it will be possible to establish patterns of occupational risk prevention. The keywords that appeared most frequently in the selected articles were T-Pattern directly associated with THEME and to a lesser extent the keyword T-String, probably because this analysis is implicit in TPA. Sentiment analysis, using Atlas.ti 9, indicated the content of the articles in terms of positive, negative, and neutral sentences, providing information about the possible results of each study. Negative sentences were more frequent, although positive sentences were found in all of the articles. This may be due to difficulties in applying this technology, which are probably related to the training users need in order to be able to apply it properly. This indicates the need for researchers to be trained in the use of these tools. Similarly, the analysis of co-occurrence and network analysis applied to the categorization of the selected articles provides a visual map that will allow researchers to select the documents that best fit their research objectives.

Finally, we would like to emphasize that applying a mixed methodology to a systematic review study allowed us to analyze the state of the art in the chosen topic, providing a great deal of quantitative and qualitative information that may be very useful to future researchers from various research perspectives (Sáiz-Manzanares et al., 2020).

## Data Availability Statement

The original contributions presented in the study are included in the article/Supplementary Material, further inquiries can be directed to the corresponding author/s.

## Author Contributions

MS-M and LA-M: design and initial writing. MS-M, LA-M, and RM-S: review and final writing. All authors contributed to the article and approved the submitted version.

## Funding

This work was funded through the Asistentes de voz e inteligencia artificial en Moodle: un camino hacia una universidad inteligente-SmartLearnUni- Project selected in the 2020 round of the Spanish Ministerio de Ciencia e Innovación (Proyectos de I+D+i - RTI Tipo B. Reference: PID2020-117111RB-I00).

## Conflict of Interest

The authors declare that the research was conducted in the absence of any commercial or financial relationships that could be construed as a potential conflict of interest.

## Publisher's Note

All claims expressed in this article are solely those of the authors and do not necessarily represent those of their affiliated organizations, or those of the publisher, the editors and the reviewers. Any product that may be evaluated in this article, or claim that may be made by its manufacturer, is not guaranteed or endorsed by the publisher.
