# Supplementary material for: A Systematic Review of the Use of T-Pattern and T-String Analysis (TPA) With Theme: An Analysis Using Mixed Methods and Data Mining Techniques
Source: Front Psychol. 2022 Jul 22;13:943907. doi: 10.3389/fpsyg.2022.943907 (PMC9354046; doi:10.3389/fpsyg.2022.943907)
Supplement: Supplementary file 1 [file Table_1.docx]

Table S1. List of articles on T-patterns, T-string and Theme in the last 4 years.

|  | Article | Keyword | | | Context | Activity | Research methods |
| --- | --- | --- | --- | --- | --- | --- | --- |
|  |  | 1 | 2 | 3 |  |  |  |
| 1 | Pic, M., Navarro-Adelantado, V., and Jonsson, G. K. (2021). Exploring playful asymmetries for gender-related decision-making through T-pattern analysis. *Physiology & behavior*. 236: 113421. doi: 10.1016/j.physbeh.2021.113421 | X | X | - | Research  Gamification | Serious games in students in secondary education | Mixed methods |
| 2 | Pic, M., Navarro-Adelantado, V., and Jonsson, G. K. (2018). Detection of Ludic Patterns in Two Triadic Motor Games and Differences in Decision Complexity. Frontiers in Psychology, 8, 2259. doi: 10.3389/fpsyg.2017.02259 | X | X |  | Research  Gamification | Serious games in students in secondary education | Mixed methods |
| 3 | Magnusson MS (2020b) T-Pattern Detection and Analysis (TPA) With THEME^TM^: A Mixed Methods Approach. *Front. Psychol. 10:* 2663. doi: 10.3389/fpsyg.2019.02663 | X | X |  | Research  Genetics | Neural Genetic Behavioural Analysis | Mixed methods |
| 4 | Casarrubea, M., Palacino, M., Brancato, A., Lavanco, G., Cannizzaro, C., Crescimanno, G. (2021). Detection of a temporal structure in the rat behavioural response to an aversive stimulation in the emotional object recognition (EOR) task. *Physiol. Behav* 238: 1–9. doi: 10.1016/j.physbeh.2021.113481 | X | X |  | Research  Treatment of  phobias | Behavioural response to an aversive stimulation | Mixed methods |
| 5 | Casarrubea, M., Di Giovanni, G., Crescimanno, G. (2021). Effects of different anxiety levels on the behavioral patternings investigated through T-pattern analysis in wistar rats tested in the hole-board apparatus. *Front. Psychol*. 11: 6. doi:10.3390/brainsci11060714 | X | X |  | Research  Emotions | Behavioural and emotional analysis in rats | Mixed methods |
| 6 | Casarrubea, M., Davies, C., Pierucci, M., Colangeli, R., Deidda, G., Santangelo, A., ... and Di Giovanni, G. (2021). The impact of chronic daily nicotine exposure and its overnight withdrawal on the structure of anxiety-related behaviors in rats: Role of the lateral habenula. Progress in Neuro-Psychopharmacology and Biological Psychiatry, 105, 110131. doi: 10.1016/j.pnpbp.2020.110131. | X | X |  | Research  Treatment of  Therapies | Behavioural and emotional analysis in rats | Mixed methods |
| 7 | Casarrubea, M., Di Giovanni, G. (2020). Application of T-pattern analysis in the study of the organization of behaviour. *Physiol. Behav.* 227: 11313. doi: 10.1016/j.physbeh.2020.113138 | X | X |  | Research  Human Behavioural Analysis | Behavioural analysis in humans | Mixed methods |
| 8 | Magnusson, M. S. (2020a). T-patterns, external memory and mass-societies in proteins and humans: In an eye-blink the naked ape became a string-controlled citizen. *Physiology & Behavior*. 227: 113146. doi: 10.1016/j.physbeh.2020.113146 | X | X | X | Research  Human Behavioural Analysis | Behavioural analysis  Neurological analysis in humans | Mixed methods |
| 9 | Brill, M., Schwab, F. (2020). T-pattern analysis and spike train dissimilarity for the analysis of structure in blinking behavior. *Physiol. Behav*. 227: 113163. doi: 10.1016/j.physbeh.2020.113163 |  | X |  | Research  Psychophysiology | Analysis of blinking in university students | Experimental method |
| 10 | Prieto-Lage, I., Louzao-Neira, I., Argibay-González, J.C., Gutiérrez-Santiago, A. (2020). Injury patterns of professional footballers in the Spanish first division during the 2017–2018 season. *Physiol. Behav*. 224: 1-10. doi: 10.1016/j.physbeh.2020.113052 | X | X |  | Research  Sport | Football injury analysis | Mixed methods |
| 11 | Gunst, N., Casarrubea, M., Vasey, P.L., Leca, J.-B. (2020). Is female-male mounting functional? An analysis of the temporal patterns of sexual behaviors in Japanese macaques. *Physiol. Behav.* 223: 1–8. doi: 10.1016/j.physbeh.2020.112983 | X | X |  | Research  Sexuality in human and non-human primates | Analysis of sexual behaviours in human and non-human primates | Mixed methods |
| 12 | Cenni, C., Casarrubea, M., Gunst, N., Vasey, P.L., Pellis, S.M., Wandia, I.N., Leca, J.-B. (2020). Inferring functional patterns of tool use behavior from the temporal structure of object play sequences in a non-human primate specie*. Physiol. Behav* 222: 1–7. doi: 10.1016/j.physbeh.2020.112938 | X | X |  | Research  Sexuality in non-human primates | Analysis of sexual behaviour in primates | Mixed methods |
| 13 | Santoyo, C., Jonsson, G. K., Anguera, M. T., Portell, M., Allegro, A., Colmenares, L., et al. (2020). T-patterns integration strategy in a longitudinal study: a multiple case analysis. *Physiol. Behav*. 222:112904. doi: 10.1016/j.physbeh.2020.112904 | X | X |  | Research  Behavioural analysis  Early Childhood Education | Behavioural analysis in preschool | Mixed methods |
| 14 | Santangelo, A., Monteleone, A.M., Casarrubea, M., Cassioli, E., Castellini, G., Crescimanno, G., Aiello, S., Ruzzi, V., Cascino, G., Marciello, F., Ricca, V. Recurring sequences of multimodal non-verbal and verbal communication during a human psycho-social stress test: A temporal pattern analysis. Physiol. (2020). *Behav.* 221: 112907. doi: 10.1016/j.physbeh.2020.112907 | X | X |  | Research  Behavioural research in university students | Behavioural analysis in university students | Mixed methods |
| 15 | Camerino, L., Camerino, O., Prat, Q., Jonsson, G.K., Castañer, M. (2020). Has the use of body image in advertising changed in the first two decades of the new century? Physiol. *Behav*. 220: 112869. doi: 10.1016/j.physbeh.2020.112869 | X | X |  | Research  Sport | Image analysis of sports footage | Mixed methods |
| 16 | Casarrubea, M., Faulisi, F., Raso, G., Aiello, S., Crescimanno, G. (2020). Early alterations of the behavioural structure of mice affected by Duchenne muscular dystrophy and tested in open-field. *Behav. Brain Res*. 386: 112609. doi: 10.1016/j.bbr.2020.112609 | X | X |  | Research  Pathologies | Behavioural analysis in mice with symptoms of Duchenne muscular dystrophy (DMD). | Mixed methods |
| 17 | Castañer, M., Aiello, S., Prat, Q., Andueza, J., Crescimanno, G., Camerino, O. (2020). Impulsivity and physical activity: A T-Pattern detection of motor behavior profiles Physiol. *Behav* 219: 112849. doi: 10.1016/j.physbeh.2020.112849 | X | X |  | Research  Sport | Behavioural analysis in non-professional athletes | Mixed methods |
| 18 | Gutiérrez-Santiago, A., Pereira-Rodríguez, R., Prieto-Lage, I. (2020). Detection of the technical and tactical motion of the scorable movements in taekwondo. *Physiol. Behav*. 217: 112813. doi: 10.1016/j.physbeh.2020.112813 | X | X |  | Research  Sport | Behavioural analysis in taekwondo athletes | Mixed methods |
| 19 | Prieto-Lage, I., Rodríguez-Souto, M., Prieto, M.A., Gutiérrez-Santiago, A. (2020). Technical analysis in Tsuri-goshi through three complementary observational analysis. *Physiol. Behav*. 216: 112804. doi: 10.1016/j.physbeh.2020.112804 | X | X |  | Research  Sport | Behavioural analysis in university judo athletes | Mixed methods |
| 20 | Casarrubea, M., Aiello, S., Santangelo, A., Di Giovanni, G., and Crescimanno, G. (2019). Different representation procedures originated from multivariate temporal pattern analysis of the behavioral response to pain in wistar rats tested in a hot-plate under morphine. *Brain Sci*. 9: 223. doi: 10.3390/brainsci9090233 | X | X |  | Research  Pharmacology | Effects of morphine use vs. placebo use in rats  Analysis of behavioural patterns | Mixed methods |
| 21 | Casarrubea, M., Di Giovanni, G., Crescimanno, G., Rosa, I., Aiello, S., Di Censo, D., Ranieri, B., Santangelo, A., Busatta, D., Cassioli, E., Galante, A., Alecci, M. and Florio, T.M. (2019). Effects of Substantia Nigra pars compacta lesion on the behavioral sequencing in the 6-OHDA model of Parkinson's disease. *Behav. Brain Res*. 362: 28-35. doi: 10.1016/j.bbr.2019.01.004 | X | X |  | Research  Pharmacology | Temporal characteristics and sequential organisation of behaviour in rats after unilateral SNC lesioning with 6-OHDA | Mixed methods |
| 22 | Casarrubea, M., Magnusson, M.S., Anguera, M.T., Jonsson, G.K., Castañer, M., Santangelo, A., Palacino, M., Aiello, S., Faulisi, F., Raso, G., Puigarnau, S., Camerino, O., Di Giovanni, G., Crescimanno, G (2021). T-pattern detection and analysis for the discovery of hidden features of behaviour. J. *Neurosci. Methods*. 310: 24–32. 10.1016/j.jneumeth.2018.06.013 | X | X |  | Research  Behaviour | Behavioural analysis in humans and non-humans | Mixed methods |
| 23 | Karcioglu, A.A., and Bulut, H. (2021). The WM-q multiple exact string matching algorithm for DNA sequences. *Comput. Biol. Med*. 136: 104656. doi: 10.1016/j.compbiomed.2021.104656. |  | X | X | Genetic research | Biomarker studies in humans | Quantitative methods |
| 24 | Anguera M. T., Portell, M., Chacón-Moscoso, S., and Sanduvete-Chaves, S. (2018). Indirect observation in everyday contexts: concepts and methodological guidelines within a mixed methods framework. *Front. Psychol.* 9:13. doi: 10.3389/fpsyg.2018.00013 | X | X |  | Research  on human behaviour | Description of observation and behavioural analysis tools | Mixed methods |
| 25 | Arias-Pujol, E., and Anguera, M.T. (2020). A Mixed Methods Framework for Psychoanalytic Group Therapy: From Qualitative Records to a Quantitative Approach Using T-Pattern, Lag Sequential, and Polar Coordinate Analyses. . *Front. Psychol. 11*: 1922. doi: 10.3389/fpsyg.2020.01922 | X | X |  | Research  Psychophysiology | The communication strategies used by a lead therapist in the initial and final phases of therapy with adolescents | Mixed methods |
| 26 | Casarrubea, M., Magnusson, M. S., Anguerac, M. T., Jonssonb, G. K., Castañer, M., Santangelo, A., et al. (2018). T-pattern detection and analysis for the discovery of hidden features of behavior. *J.* Neurosci. Methods 310: 24–32. doi: 10.1016/j.jneumeth.2018.06.013 | X | X |  | Research  Human behaviour | Use of THEME software for behavioural analysis in different species | Mixed methods |
| 27 | Portell, M., Sene-Mir, A., Anguera, M.T., Jonsson, G.K., and Losada J.L. (2019). Support system for the assessment and intervention during the manual material handling training at the workplace: contributions from the systematic observation. *Front. Psychol. 10*: 1247. doi: 10.3389/fpsyg.2019.01247. | X | X |  | Research  Human behaviour | Study on health promotion in the workplace | Mixed methods |
| 28 | Suárez, N., Sánchez, C. R., Jiménez, J. E., and Anguera, M. T. (2018). Is reading instruction evidence-based? Analyzing teaching practices using T-Patterns. *Front. Psychol. 9:*7. doi: 10.3389/fpsyg.2018.00007 | X | X |  | Research  Human behaviour | Study of the teaching-learning process in Early Childhood Education. | Mixed methods |
| 29 | Casarrubea, M., Aiello, S., Di Giovanni, G., Santangelo, A., Palacino, M., and Crescimanno, G. (2019). Combining quantitative and qualitative data in the study of feeding behavior in male wistar rats. *Front. Psychol.* 10: 881. doi:10.3389/fpsyg.2019.00881 | X | X |  | Animal behaviour research | Behavioural analysis of rats on standard vs. hyperglycaemic diet | Mixed methods |
| 30 | Del Giacco, L., Salcuni, S., and Anguera, M. T. (2019). The communicative modes analysis system in psychotherapy from mixed methods framework: introducing a new observation system for classifying verbal and non-verbal communication. *Front. Psychol.* 10:782. doi: 10.3389/fpsyg.2019.00782 |  | X |  | Research  Human behaviour | Analysis of verbal and non-verbal behaviours in psychotherapy situations | Mixed methods |
